# Cancer Patients’ Use of Apitherapy as Supportive Care: An Exploratory Study

**DOI:** 10.5152/eurasianjmed.2024.22185

**Published:** 2024-02-01

**Authors:** Mehtap Kavurmacı, İdris Yıldız

**Affiliations:** Department of Internal Medicine Nursing, Atatürk University Nursing Faculty, Erzurum, Turkey

**Keywords:** Apitherapy, complementary and alternative medicine, cancer, oncology, supportive care

## Abstract

**Background:**

This study was conducted to determine the cancer patients’ use of apitherapy as supportive care.

**Methods:**

This descriptive research was carried out between January 2019 and January 2020 at the oncology unit of a university hospital. Power analysis was used to determine the sample size of the study, and a total of 87 patients constituted the sample of the research with a CI of 85%. A questionnaire consisting of questions investigating the sociodemographic characteristics of the patients and their knowledge, opinions, and practices about apitherapy was used to collect the data. The data were analyzed using descriptive statistics and the Statistical Package of Social Sciences 21 package program.

**Results:**

The level of knowledge of the patients about apitherapy is quite low (41.4%), and they received the information they gained from their family/friends (63.9%). The rate of use of apitherapy products by patients is low (27.6%), and the most frequently used product is honey (37.4%). Patients stated that apitherapy products should be used to support cancer treatment (57.5%) and should be used in consultation with a physician (54.0%). The patients who use apitherapy products the most are those with breast cancer.

**Conclusion:**

In order for apitherapy to be used as a support in cancer treatment, it is important for healthcare professionals to guide patients about the areas of use of apitherapy and its safe use.

**Main Points**

- Oncology patients have a low level of knowledge about the use of apitherapy.
- The most preferred apitherapy product by patients is honey.
- Patients’ attitudes toward apitherapy products are generally positive but they need more scientific evidence.
- Patients want apitherapy products to be more accessible and cheap.
- The patients who use apitherapy products the most are those with breast cancer.

## Introduction

Cancer is an increasingly common health problem all over the world, and it is difficult to treat. Every year, millions of people around the world are diagnosed with cancer, and more than half of these people die.^1^ Many people who are diagnosed with cancer feel helpless and seek different treatment methods. Complementary and alternative medicine (CAM) practices are one of the treatment methods that arouse curiosity among cancer patients and that some choose to try. Today, the use of CAM practices is very common among cancer patients, and its rate of use increases day by day.^2^ Cancer patients may choose to use CAM for purposes such as supporting their regular treatment, eliminating the side effects of chemotherapy, relieving symptoms, relaxing physically and mentally, strengthening the immune system, and preventing the recurrence of cancer.^3-7^ Complementary and alternative medicine practices that are frequently used by cancer patients are herbal products, relaxation, hypnosis, acupuncture, acupressure, yoga, meditation, massage, music, reflexology, cryotherapy, aromatherapy, and apitherapy.^2,8-12^

Apitherapy is a CAM method in which honeybee products are used for therapeutic purposes. In addition to being used as nutrients, apitherapy products have also been used for the treatment of diseases throughout history due to the substances they contain that have biologically active properties. The most commonly used apitherapy products are honey, propolis, pollen, beeswax, royal jelly, and bee venom.^13,14^ There are many studies in the literature reporting that apitherapy products have antimicrobial, antioxidant, anti-inflammatory, immunomodulatory, and anticancer effects.^15-17^

In order for these beneficial effects of apitherapy products to be used safely for therapeutic purposes, healthcare professionals should conduct research, provide reliable information, and guide patients correctly. Although there are studies in the literature that examine the use of general CAM practices in cancer patients,^7-12^ there are no studies specifically examining the use of apitherapy products. In this context, the general information, sources of information, and prejudices of oncology patients about apitherapy and the apitherapy products they use are subjects that have not been clarified yet. This study was conducted to determine the cancer patients’ use of apitherapy as supportive care.

### Research questions

- What is the use of apitherapy products by cancer patients?
- What are the apitherapy products used by cancer patients?
- What are the opinions of cancer patients about the use of apitherapy products?

## Material and Methods

### Objective and Design

The descriptive research was conducted in a university hospital located east of Turkey between January 2019 and January 2020.

### The Population and Sampling of the Research

The population of the research consisted of 102 patients who were treated in the oncology unit on the relevant dates. Patients who were 18 years of age or older and had been receiving cancer treatment for at least 6 months were included in the research sample.Power analysis was used to determine the sample size of the study and a total of 87 patients were included in the study with a confidence interval of 85%.

### Data Collection Tools

A questionnaire developed by the researchers was used to collect the data, which consisted of questions that investigated the sociodemographic characteristics of the patients and their knowledge, opinions, and practices about apitherapy.

#### The Questionnaire Form

The questionnaire consists of 2 parts. In the first part, there are 9 questions regarding the sociodemographic and disease characteristics of the participants (e.g., age, gender, marital status, medical diagnosis, and disease duration). In the second part, there are 16 questions to determine the knowledge, opinions, and practices of the patients about apitherapy.^18,19^

The questionnaires were filled by the patients. It took approximately 10-15 minutes to complete each questionnaire. The completed questionnaires were collected back by the researcher.

### Data Evaluation

For statistical analysis, International Business Machines Statistical Package of the Social Sciences (SPSS) v.22 (IBM SPSS Corp., Armonk, NY, USA) was used. Descriptive (percentage, arithmetic mean, SD, and minimum–max) tests were used in the analysis.

### Ethical Dimension of the Research

Before starting the research, ethics committee approval was obtained from Atatürk University Clinical Research Ethics Committee (Approval no: 2018-12/12, Date: 5 December, 2018), and written permissions were obtained from the hospital where the research would be conducted. After the patients participating in the study were informed about the study, verbal and written consents of the participants who agreed to participate in the study were obtained.

### Limitations of the Research

Conducting research only with patients in the oncology department of the relevant hospital and on the specified dates is the limitation of the research.

## Results

Thirty-one percent of patients were between the ages of 44 and 56 years, and 54% were male, 86.2% were married, 41.4% were housewives, 51.7% were literate or primary school graduates, 65.5% had a medium level of income, and 65.5% lived in the city center. The disease duration of 93.1% of the patients was 0-5 years, the medical diagnosis of 26.4% of the patients was breast cancer, and the treatment method of 77% of the patients was chemotherapy (Table 1).

**Figure 1.**
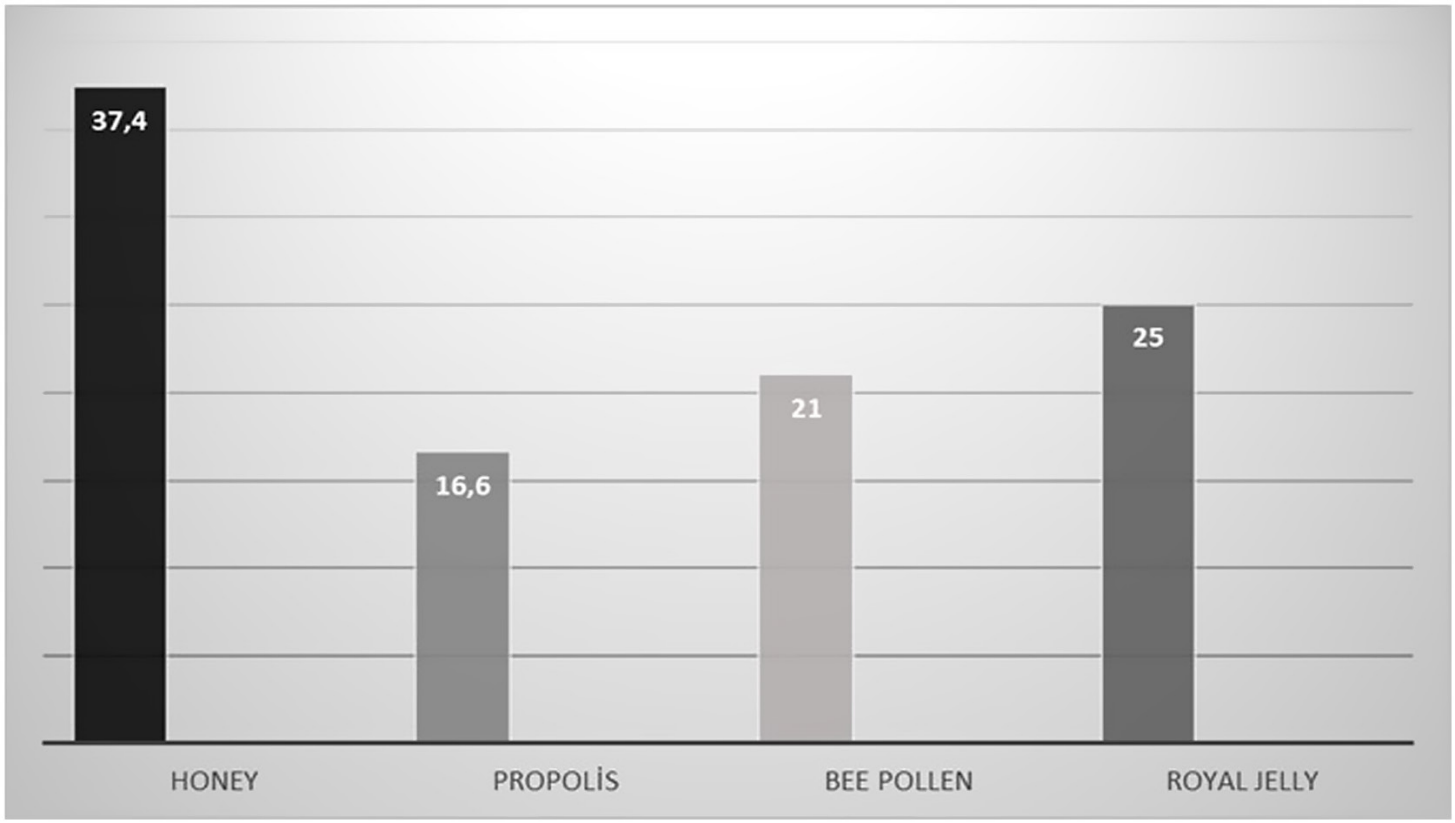
Distribution of apitherapy products used by patients.

Only 41.4% of the patients had knowledge about apitherapy, and 63.9% received this knowledge from their family/friends. About 27.6% of the patients used apitherapy products, but only 25% of them got the approval of their physician to use these products. About 54.2% thought that the apitherapy products they use were beneficial to them (Table 2). About 37.4% of the participants stated that they used honey, 25% used royal jelly, 21% used bee pollen, and 16.6% used propolis. None of the participants used bee bread or bee venom (Figure 1).

Of the patients (72.4%) who did not use apitherapy products, 41.3% stated that they do not find these products reliable, 23.8% stated that they found them expensive and they were afraid of the side effects, and 11.1% stated that they do not believe apitherapy products have any benefits (Table 2).

**Figure 2.**
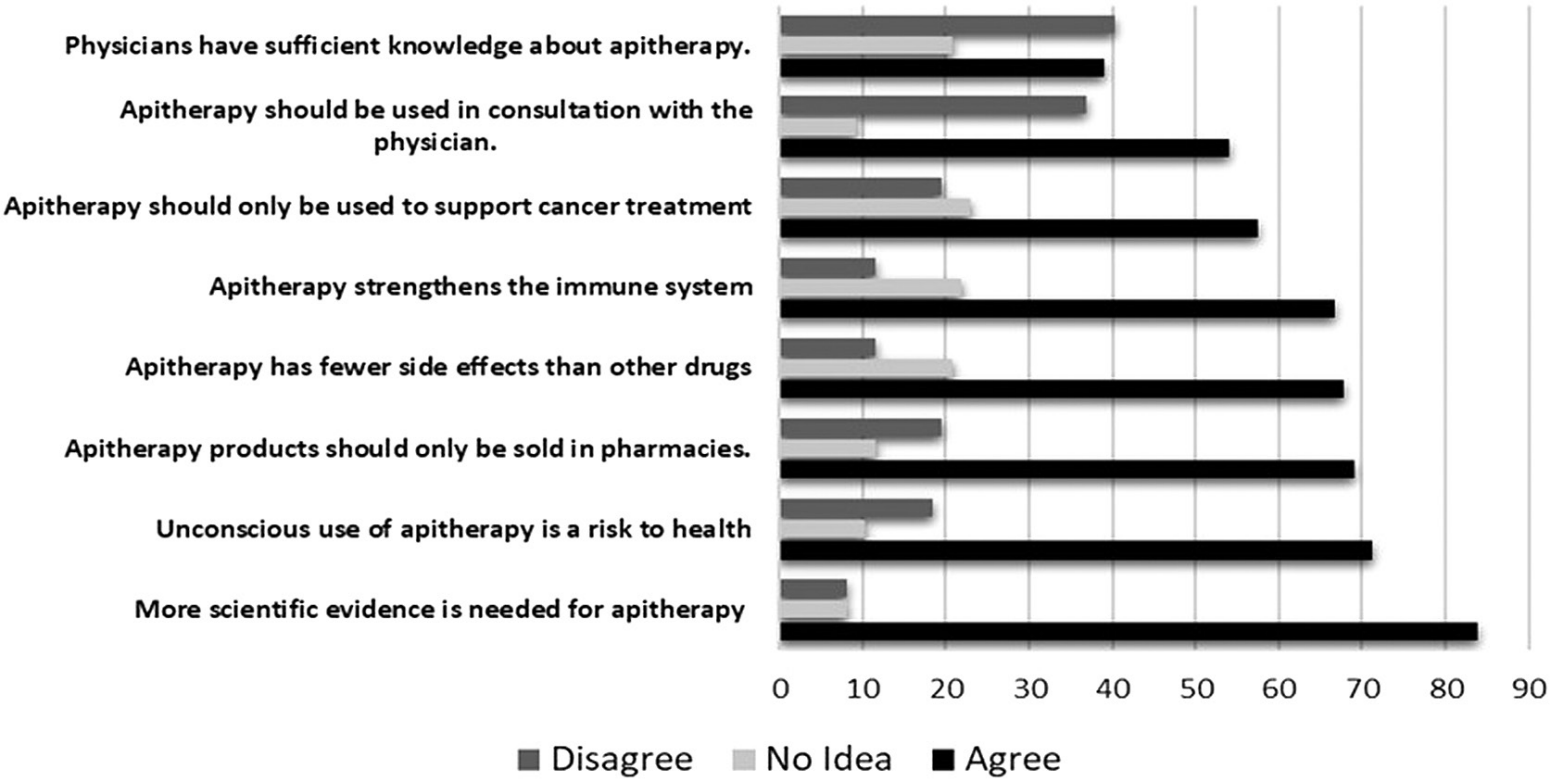
Distribution of patients’ opinions on the use of apitherapy products as a support in cancer treatment.

In Figure 2, 83.9% of the patients opinion that more scientific evidence is needed for apitherapy products to be used in cancer treatment. Similarly, it was determined that patients thought that unconscious use of apitherapy products would pose a risk to public health (71.3%) and that these products should be sold only in pharmacies (69%). Patients stated that apitherapy products should only be used to support cancer treatment (57.5%) and should be used in consultation with a physician (54.0%). On the other hand, 40% of patients think that physicians do not have enough knowledge about apitherapy.

**Figure 3.**
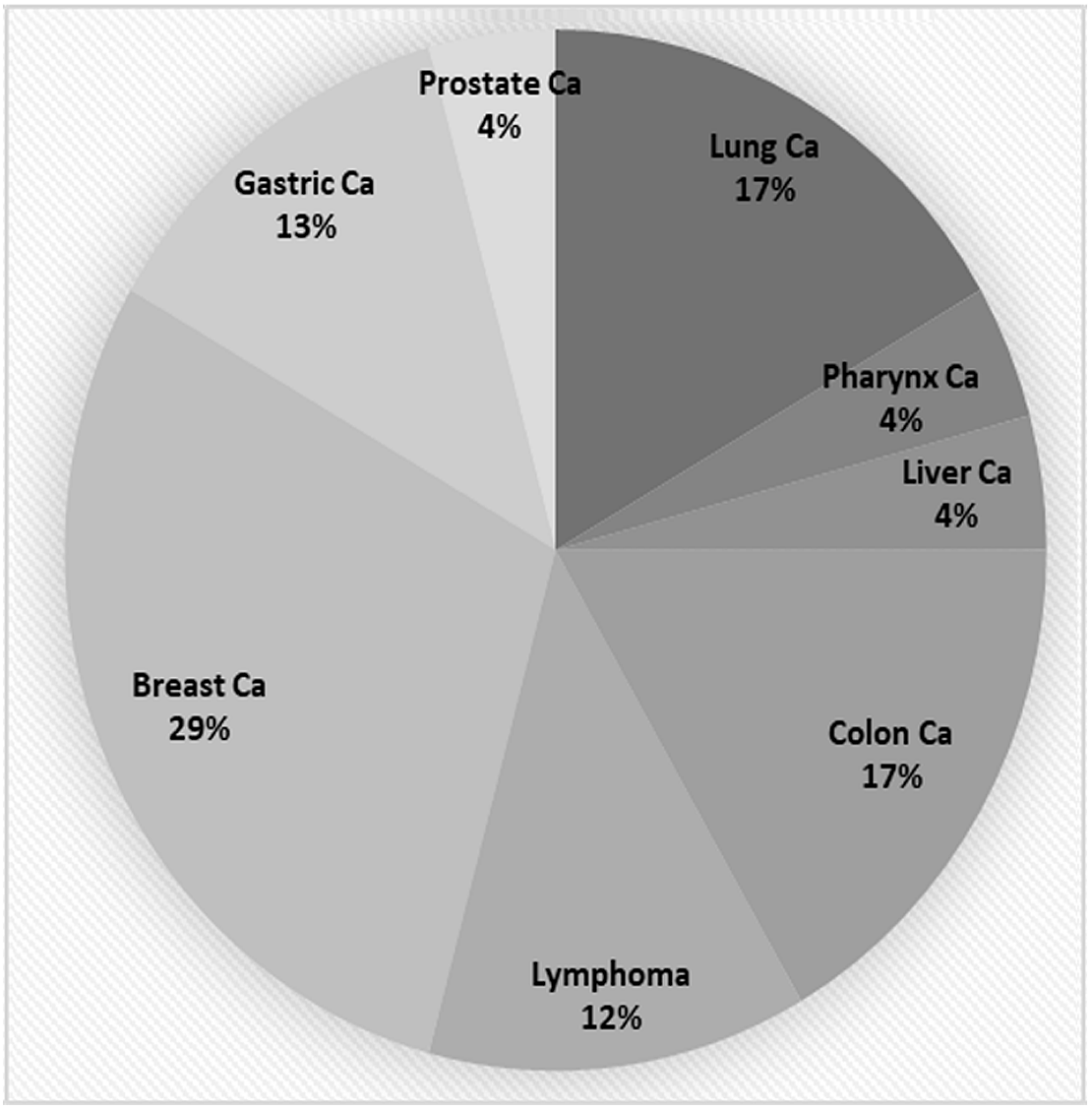
Use of apitherapy products according to medical diagnosis.

When the use of apitherapy products was compared according to the medical diagnosis of the patients, it was determined that the patient group using apitherapy products the most was the group of patients with breast cancer (Figure 3).

## Discussion

Apitherapy products are among the CAM practices that are commonly used in cancer treatment, and it is thought that the anticancer effect of these products is through apoptosis, necrosis, and lysis of tumor cells.^15^ It is reported in the literature that apitherapy products have anticancer effects on various tumor cells such as breast cancer, cervical cancer, leukemia, kidney cell cancer, bladder cancer, colon cancer, prostate cancer, and oral cancer.^20-33^ Although there are a limited number of studies demonstrating that bee pollen and royal jelly are therapeutic or preventive for cancer, it has been reported that it has a palliative effect in cancer patients.^15^

In our study, the first finding that draws attention is the knowledge level of the patients about apitherapy. It was found that only 41.4% of the patients participating in the study had information about apitherapy and 63.9% received this information from their family/friends (Table 1). When the literature is examined, no study has been found that specifically examines oncology patients’ knowledge and use of apitherapy products. However, there are many studies in the literature that examine the knowledge and use of CAM practices of oncology patients in general, and it has been found that the patients generally have a moderate knowledge of CAM and they get this information from their family/friends.^3-6,30^ Münstedt et al,^29^ on the other hand, in their study conducted with the general patient population who applied to the family doctor or gynecologist for control, found that 1.4% of the patients had sufficient knowledge about apitherapy, 2.1% had some knowledge about apitherapy, 19% had very little knowledge, and 77.5% had no knowledge. It is pleasing that in our study, the knowledge level of oncology patients about apitherapy was higher than the results of Münstedt et al.^29^ However, it is concerning that patients mostly received their knowledge about apitherapy from family/friends. Unfortunately, apitherapy products are CAM practices that are used either by hearing about them from someone or by empirical approaches in the society, and this situation prevents patients from using apitherapy effectively and correctly. However, if healthcare professionals equipped with apitherapy knowledge inform and guide the patients, potential risks will be reduced, and apitherapy will be used effectively and correctly. Nevertheless, in studies examining the knowledge and attitudes of healthcare professionals about apitherapy and other CAM practices, it was found that the knowledge level of healthcare professionals was not at the desired level either.^28,34-38^ These results demonstrate that first healthcare professionals and then oncology patients should be informed about apitherapy and other CAM practices.

As a result of the study, it was found that only 27.6% of the patients used apitherapy products, and 72.4% did not use the apitherapy products for reasons such as finding them unreliable/expensive (41.3%), being afraid of side effects (23.8%), and not believing that they are beneficial (11.1%). It was found that 54.2% of the patients using apitherapy benefit from apitherapy products, but only 25% of them got approval from their physician before using these products. There is no study in the literature indicating the rate of use of apitherapy products by oncology patients, so the data obtained from this study are pioneering. However, it is known that oncology patients generally use CAM practices at a rate of 46%-61.2%, and most of them do not get approval from their physicians before using these methods, similar to the results obtained in our study.^3-5,30^ When the apitherapy products used by the patients are examined, it is seen that 37.4% use honey, 25% use royal jelly, 21% use bee pollen, and 16.6% use propolis (Figure 1 and Table 2). Münstedt et al^29^ found that the most widely known apitherapy products are honey and propolis and that patients are reluctant to use apitherapy products other than honey for medical purposes. Honey is a bee product that is widely known and consumed by people as a nutrition/energy source and for therapeutic purposes because of its high energy and carbohydrate content, taste, aroma, and other superior properties.^24^ There are many studies in the literature reporting that honey inhibits cancer cells due to its bioactive components such as phenolic acid and flavonoids and that these compounds inhibit the cancer-causing free radical formation and oxidative stress.^15,20,32^ Royal jelly, bee pollen, and propolis, which are the most widely known bee products after honey, have many biological effects ranging from antimicrobial, antioxidant, anti-inflammatory, immunomodulatory, and anticancer effects.^18,19,23^ Honey is a natural food, and it is more available than other bee products such as propolis, royal jelly, bee venom, beeswax, and pollen.^33^ We believe that the low rate of an apitherapy product use of patients is due to the fact that bee products other than honey are not well known by society and that honey is consumed as a nutrient rather than for medical purposes. Honey is widely consumed as a nutrient in Turkey, and this geography is suitable for beekeeping. As of 2016, approximately 7 900 364 hives and 105 727 tons of honey were produced.^34^

In our study, the attitudes of the patients about the use of apitherapy products and risks were also examined, and it was found that 55.2% of the patients believe that those with allergies should not use apitherapy products and 16.1% believe that babies under 1 year should not use them (Table 2). Since bee venom, one of the apitherapy products, may cause allergic reactions and death, it should be used very carefully.^21^ On the other hand, the use of honey in babies younger than 1-year-old is not recommended as it may cause botulism and allergic reactions.^13^ Our results show that the knowledge level of the patients about the allergic risks of apitherapy products is not sufficient. In order for patients to use apitherapy products safely, they need to be informed about the mechanism, potential risks, and age groups of these products, and healthcare professionals have important responsibilities in this regard.

About 48.3% of the patients stated that they see apitherapy as a popular CAM method, 57.5% think that it should be used to support cancer treatment, 59.8% think that it should be widespread among cancer patients, 66.7% think that it supports the immune system, 67.8% think that it has fewer side effects than medical treatment. On the other hand, 83.9% of the patients stated that they think that more scientific evidence is needed for the use of apitherapy products in the treatment of diseases and 69% of them find apitherapy products sold in the market expensive and do not believe they are reliable (Figure 2). In their study, Münstedt et al^29^ found that patients only see honey as an acceptable treatment method and that they did not find products such as bee venom reliable. The results show that although patients generally have a positive attitude toward apitherapy products, they need more scientific evidence, and these products need to be more accessible and economical for patients to use them. For this purpose, we believe that it will be beneficial to increase the number of studies aimed at determining the knowledge, attitude, and practices of patients with chronic diseases such as cancer.

According to the results, patients with breast cancer use apitherapy products more (Figure 3). Complementary and alternative medicine therapy is widely used in patients diagnosed with breast cancer.^39^ In the study of Bebiş et al,^40^ the frequency of using CAM in breast cancer patients was found to be between 24% and 98%. There are studies in the literature reporting that apitherapy products, and especially honey, are beneficial in the treatment of breast cancer.^41-43^ Kurt et al^44^ found that 15.8% of breast cancer patients used honey and 7.9% used royal jelly and pollen in their studies.

The results show that patients should be informed, guided, and followed up by specialist healthcare professionals in order to increase their knowledge about apitherapy. However, unfortunately, the number of healthcare professionals who are knowledgeable, well-trained, and specialized in apitherapy is very low, both in our country and in the world. For this reason, in order to actively use existing units in hospitals, CAM units should be established and health professionals who are experts in the field of apitherapy should be trained in these units.

In addition to these results, it was found that oncology patients need more scientific evidence in order to see apitherapy as a reliable treatment method. For this purpose, we propose more scientific research on the subject and repeating these studies in different geographical regions and wider populations. We believe that the results of this research will increase the reliability, recognition, and usage rates of apitherapy products and make these products more accessible and economical.

## Figures and Tables

**Table 1.** Patients’ Demographic Characteristics

	n	%
Age	3	3
18-30 years	7	8.0
31-43 years	11	12.6
44-56 years	27	31.0
57-69 years	26	29.9
70 years and above	16	18.4
Gender	3	3
Female	40	46.0
Male	47	54.0
Marital status	3	3
Married	75	86.2
Single	12	13.8
Employment status	3	3
Worker/officer	19	21.8
Retired	20	23.0
Housewife	36	41.4
Self-employed	12	13.8
Economic status	3	3
Good	14	16.1
Average	57	65.5
Bad	16	18.4
Educational status	3	3
Illiterate	10	11.5
Literate/primary school	45	51.7
Middle school/high school	24	27.6
Associate degree/bachelor degree	8	9.2
Medical diagnosis	3	3
Breast cancer	23	26.4
Lung cancer	14	16.1
Colon cancer	13	14.9
Lymphoma	12	13.8
Gastric cancer	11	12.6
Prostate cancer	6	6.9
Pharynx cancer	4	4.6
Liver cancer	4	4.6
Duration of diseases (years)	3	3
0-5 years	81	93.1
6-11 years	6	6.9
Method of treatment	3	3
Chemotherapy	67	77.0
Chemotherapy + radiotherapy	20	23.0

**Table 2.** The Patients’ Level of Knowledge About Apitherapy and Their Use of the Products

	n	%
The state of knowing about apitherapy (n = 87)	3	3
Yes	36	41.4
No	51	58.6
Source of knowledge about apitherapy (n = 36)	3	3
Friends/other family members	23	63.9
Internet	7	19.4
Doctor/nurse	6	16.7
Beekeeper in the family (n = 87)	3	3
Yes	17	19.5
No	70	80.5
Status of using apitherapy products (n = 87)	3	3
Yes	24	27.6
No	63	72.4
Getting permission from the doctor to use apitherapy (n = 24)	3	3
Yes	6	25.0
No	18	75.0
Apitherapy products used (n = 24)	3	3
Honey	9	37.4
Propolis	4	16.6
Bee pollen	5	21.0
Royal jelly	6	25.0
Satisfaction with apitherapy products (n = 24)	3	3
Yes	13	54.2
No	5	20.8
A little	6	25.0
Reason for not using apitherapy products (n = 63)	3	3
Too expensive	15	23.8
Not helpful	7	11.1
Not reliable	26	41.3
Have side effects	15	23.8
